# *In Vivo* and *Ex Vivo* Evaluation of L-Type Calcium Channel Blockers on Acid β-Glucosidase in Gaucher Disease Mouse Models

**DOI:** 10.1371/journal.pone.0007320

**Published:** 2009-10-07

**Authors:** Ying Sun, Benjamin Liou, Brian Quinn, Huimin Ran, You-Hai Xu, Gregory A. Grabowski

**Affiliations:** The Division of Human Genetics, Cincinnati Children's Hospital Medical Center, and the Departments of Pediatrics, University of Cincinnati College of Medicine, Cincinnati, Ohio, United States of America; National Institutes of Health, United States of America

## Abstract

Gaucher disease is a lysosomal storage disease caused by mutations in acid β-glucosidase (GCase) leading to defective hydrolysis and accumulation of its substrates. Two L-type calcium channel (LTCC) blockers—verapamil and diltiazem—have been reported to modulate endoplasmic reticulum (ER) folding, trafficking, and activity of GCase in human Gaucher disease fibroblasts. Similarly, these LTCC blockers were tested with cultured skin fibroblasts from homozygous point-mutated GCase mice (V394L, D409H, D409V, and N370S) with the effect of enhancing of GCase activity. Correspondingly, diltiazem increased GCase protein and facilitated GCase trafficking to the lysosomes of these cells. The *in vivo* effects of diltiazem on GCase were evaluated in mice homozygous wild-type (WT), V394L and D409H. In D409H homozygotes diltiazem (10 mg/kg/d via drinking water or 50–200 mg/kg/d intraperitoneally) had minor effects on increasing GCase activity in brain and liver (1.2-fold). Diltiazem treatment (10 mg/kg/d) had essentially no effect on WT and V394L GCase protein or activity levels (<1.2-fold) in liver. These results show that LTCC blockers had the *ex vivo* effects of increasing GCase activity and protein in the mouse fibroblasts, but these effects did not translate into similar changes *in vivo* even at very high drug doses.

## Introduction

Deficiency of GCase leads to accumulation of its substrates, glucosylceramide (GC) and glucosylsphingosine (GS), and the resultant visceral and CNS phenotypes in Gaucher disease [1], [2]. Three types of Gaucher disease are clinically delineated, based on age at recognition and organ involvement. Type 1 is the non-neuronopathic variant with highly variable visceral disease [2]. Types 2 and 3 have early onset of variable CNS deterioration [1]. About 350 mutations in GCase have been identified in DNAs from affected patients and many of these are rare or occur in single families [2]. A mutation with high frequency in the Ashkenazi Jewish population is N370S. Homozygosity for N370S is associated with type 1, non-neuropathic disease and variable visceral involvement [2], [3], [4]. The L444P recurrent mutation is highly associated with neuronopathic variants of Gaucher disease, and is the most common Gaucher disease allele world-wide [2]. The D409H alleles also has significant frequency and homozygotes manifest early onset of variable visceral and the CNS involvement [5]. Uniquely, calcific aortic root and valve disease occurs with D409H homozygosity [6]. The V394L allele in humans has been reported only in the heteroallele state and is associated with either type 1 or types 2 and 3 depending on the heteroallele [7]. In contrast to humans, N370S and L444P homozygosity in mice lead to death within 24 hours [8]. In mice homozygosity for V394L and D409H leads to defective GCase activity [8] and survival to >12 mos. with only minor visceral abnormalities [8].

Currently treatment for Gaucher disease includes enzyme replacement therapy (ERT) and substrate reduction therapy (SRT). ERT has significantly improved the health of Gaucher patients by reversing the visceral disease [9], but it has no effect on the neuronopathic manifestations due to the insufficient amounts of enzyme penetrating the blood-brain barrier (BBB) for therapeutic effect. SRT has been used with patients who cannot receive ERT, but the therapeutic index of SRT is low, and it is associated with more side effects [10]. The FDA and EMEA approved drug for SRT, miglustat, does penetrate the blood brain barrier [11]. Both ERT and SRT are expensive: >▒130,000–300,000 per year for drug costs alone. Alternative therapies are needed to access the CNS and reduce the costs.

Small molecules that modulate *in vivo* or *ex vivo* GCase stability or activity have been evaluated recently [12], [13]. For selected GCase variants, a so-called pharmacological chaperone, isofagomine (IFG), enhances GCase activity by stabilizing the enzyme in the ER or enhancing transport of mature GCases into the lysosome or both [13]. Two LTCC blockers, verapamil and diltiazem, were reported to affect ER folding, trafficking, and activity of GCase in fibroblasts from Gaucher disease patients [12], [14]. Here, these LTCC blockers were evaluated in GCase point mutated mice and derived cells for their effects on GCase activity *in vivo* and *ex vivo*.

## Results

**Figure 1:**
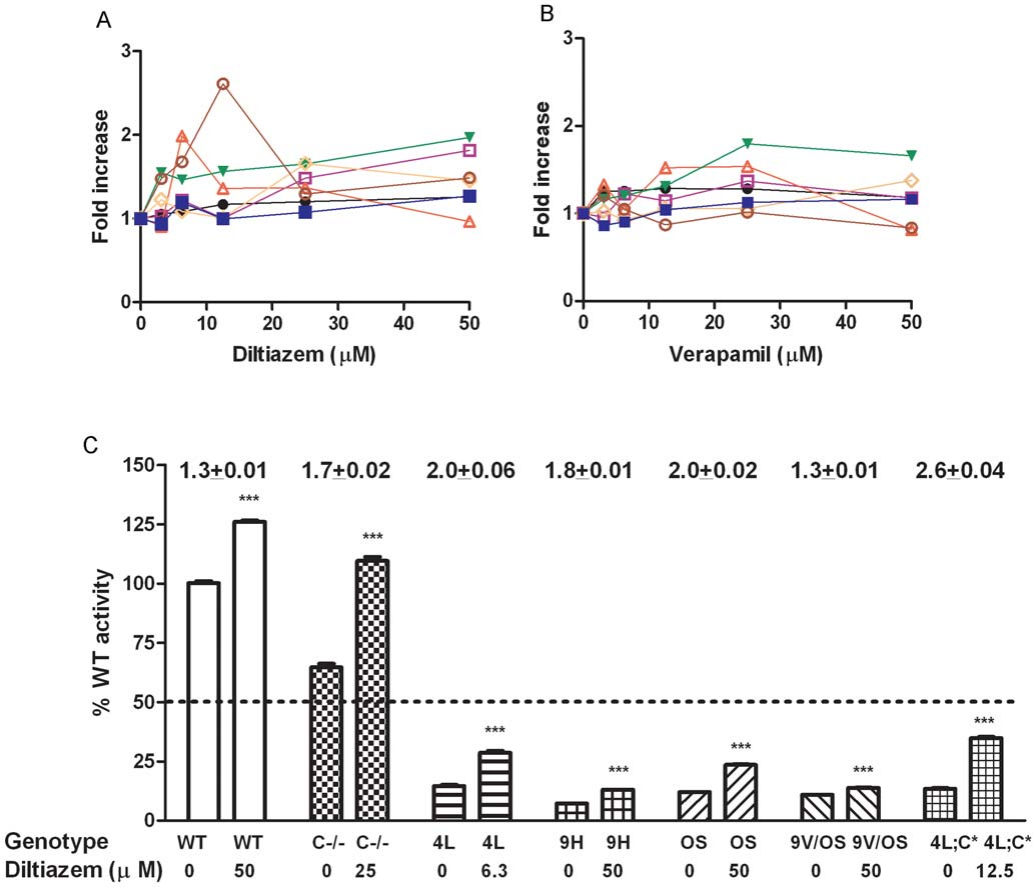
Diltiazem (A) and Verapamil (B) effects on GCase activities in fibroblasts. (A) Fold increase in GCase activities in diltiazem treated fibroblasts from WT (•), D409H homozygote (9H; □), V394L homozygote (4L; Δ), N370S homozygote (OS; ▾), 4L;C* V394L plus C−/− (4L;C*; ○), (saposin C−/−) (C−/−; ◊) and D409V/N370S compound heterozygote (9V/OS; ▪). (B) Fold increase in GCase activities in verapamil treated fibroblasts from WT, 9H, 4L, OS, 4L;C*, C−/− and 9V/OS compound heterozygotes. The cells were incubated in the presence of diltiazem or verapamil for 5 days. The enhancements of specific GCase activities in treated cells are presented as fold increase relative to the respective untreated cells. (C) Percentage of untreated WT activity in diltiazem (0 or 50 µM) treated fibroblasts. The GCase activities were significantly increased in treated fibroblasts. The activities in all treated GCase mutant cells (9H, 4L, OS, 4L;C* and 9V/OS) were below 35% of WT levels. Fold-changes (mean ± S.E.M) of diltiazem effects on GCase for each cell line are above the columns. The experiments were done in triplicates. Student's t test, ***, p<0.001.

Mouse fibroblasts were from WT, GCase point-mutated mice (V394L, D409H, and N370S homozygotes, D409V/N370S compound heterozygotes), and saposin C-deficient (C−/−) plus V394L homozygotes (4L;C*). These cells were grown to 35% confluency and incubated in a medium containing diltiazem (0 to 50 µM) for 5 days. The cell lysates were assayed for GCase activity and protein. GCase activity in V394L, D409H, N370S, C−/−, and 4L;C* fibroblasts increased ≥1.5-fold at 50 µM diltiazem in the dose-response experiments (Fig. 1A). The maximal GCase enhancements with V394L was achieved at ∼6 µM and with 4L;C* at ∼12 µM. WT and D409V/N370S fibroblasts showed a ∼1.3-fold increase in GCase activity at 50 µM. These maximal activities for each of the cell strains are displayed as the percentage of untreated WT levels (Fig. 1C). Relative to untreated WT, diltiazem-treated mutant cells achieved 13% to 35% of the WT GCase activity levels. The activities were significantly increased by 1.3- to 2.6-fold compared to untreated cells. Only diltiazem-treated C−/− cells achieved WT GCase activity levels.

A second LTCC blocker, verapamil, was tested for its effects on GCase activities from the same mouse fibroblasts (Fig. 1B). The GCase activities in verapamil-treated V394L and N370S cells showed a ≥1.5-fold activation at 25 µM. WT cells responded to verapamil with ∼1.3-fold enhancement of activity. The other cells had a ≤1.3-fold increase in GCase activity. These results indicate that both WT and variant GCases in mouse fibroblasts responded to diltiazem and verapamil. Diltiazem had more significant effects on GCase than verapamil. The enhanced activities from those treated GCase mutant were ≥35% of the WT level.

**Figure 2:**
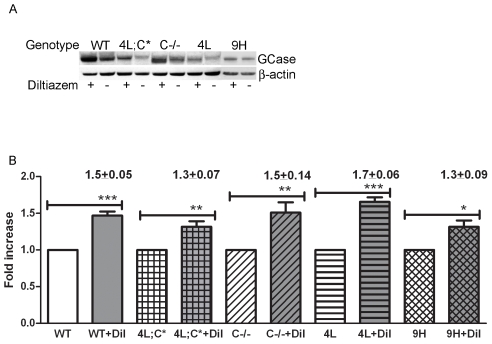
GCase protein levels in diltiazem-treated cultured fibroblasts. (A) Immunoblot of mouse GCase and β-actin protein in diltiazem (12.5 µM) treated and untreated WT, 4L, 9H, C−/− and 4L;C* cell lysates. GCase protein signal levels were increased in the diltiazem-treated cells. GCase proteins in these fibroblast extracts were detected using rabbit anti-mouse GCase antibody. β-actin was the loading control. (B) Fold changes in GCase protein signal levels in diltiazem (Dil)-treated cells relative to untreated cells. GCase protein levels were significantly increased in treated cells. Quantitation was performed using Molecular Dynamics ImageQuant 5.0 Software using the ratio of GCase/β-actin for normalization. Each experiment was repeated in triplicate. Fold-changes (mean ± S.E.M) of diltiazem effects on GCase for each cell line are above the columns. Student's t test, ***, p<0.001; **, p<0.01; *, p<0.05.

**Figure 3:**
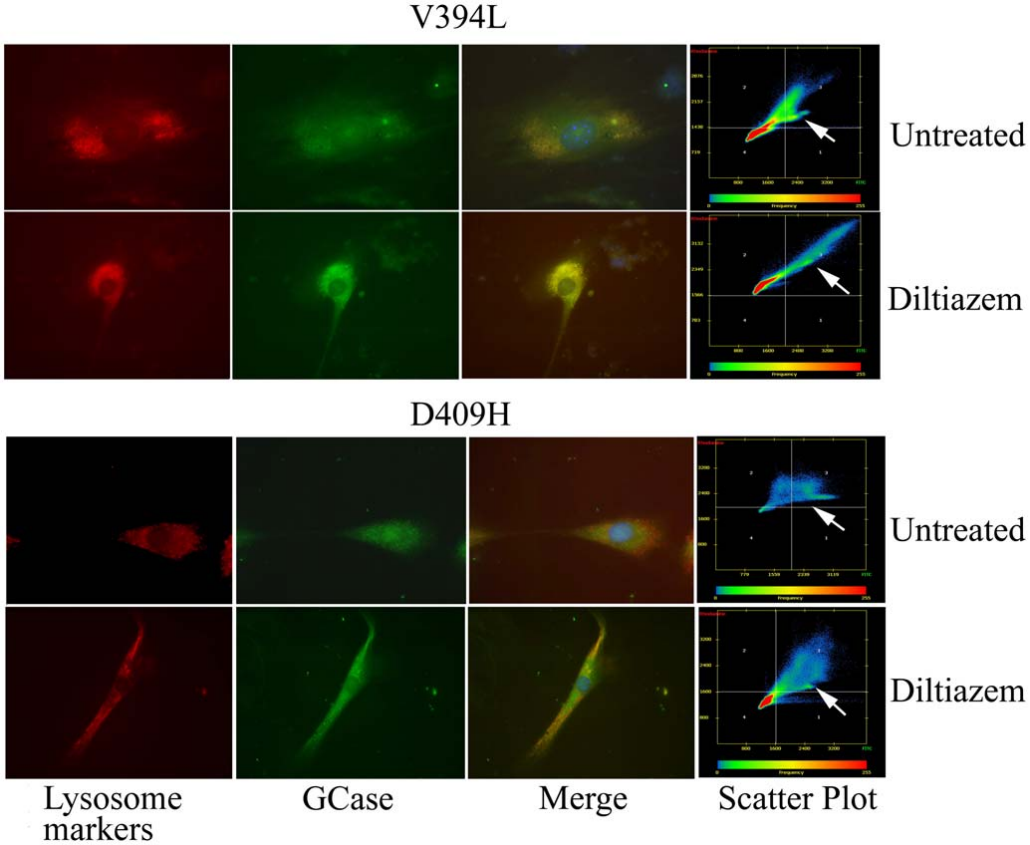
Diltiazem treatment increased localization of GCase protein in lysosome. (Top panel) Compared to untreated cells, enhanced GCase signals (green) in diltiazem (25 µM) treated mouse V394L fibroblasts colocalized with LysoTracker (Red). LysoTracker (Red) fluorescence intensities were equivalent in both treated and untreated cells. (Bottom panel) Increased D409H GCase (green) expression after diltiazem (25 µM) treatment was partially localized with Lamp1 (Red), a lysosomal marker. Most of the D409H GCase was localized to the perinuclear region before treatment. Scatter plots showed colocalization of GCase in the green FITC channel with LysoTracker or Lamp1 in the red Rhodamine channel in the upper right quadrant (arrow). For lysosome markers, LysoTracker was used for V394L cells and anti-Lamp1 was used for D409H cells.

GCase protein levels and lysosomal localization were determined in diltiazem-treated V394L, D409H, 4L;C*, C−/−, and WT cells. GCase protein levels were assessed by immuno blot analysis using rabbit anti-mouse GCase antibody. Compared to untreated cells, GCase protein levels were significantly increased by 1.3- to 1.7-fold in the treated cells with any genotype (Fig. 2). GCase trafficking to the lysosome was analyzed by colocalization quantification of fluorescent signals from goat anti-mouse GCase (FITC, green) and lysosome marker (LysoTracker or Lamp1, Red). Diltiazem (25 µM) enhanced GCase signals in cells from WT, V394L, C−/−, and 4L;C* mice (Fig. 3). Pearson's correlation coefficients were used to determine the degree of colocalization of the GCase signals with lysosomal signals (LysoTrack or Lamp1) and their change with diltizem treatment. These coefficients portray the degree of overlap between two images in FITC and Rhodamine channels and not the level of signals [15], i.e., values of 0 and 1 indicate no correlation and complete correlation, respectively. Diltiazem treatment significantly increased Pearson's correlation coefficients for GCase in WT, C−/−, D409H, V394L, and 4L;C* cells. The GCases prior to treatment were localized mostly to the perinuclear regions, whereas following treatment there were much greater lysosomal colocalization signals (Fig. 3, Table 1). These results indicate that diltiazem increases the GCase protein levels and facilitates GCase trafficking into lysosomes in cultured fibroblasts.

**Table 1:** Pearson's correlation coefficients in fibroblasts treated with diltiazem (25 µM).

Fibroblasts	Pearson's correlation coefficients*		
	− Diltiazem	+ Diltiazem	*p* value
WT	0.600±0.031 (n = 10)	0.870±0.028 (n = 13)	<0.0001
V394L/V394L	0.355±0.040 (n = 11)	0.785±0.020 (n = 18)	<0.0001
D409H/D409H	0.017±0.017 (n = 6)	0.488±0.038 (n = 11)	<0.0001
C−/−	0.528±0.033 (n = 7)	0.697±0.035 (n = 10)	<0.0001
4L;C*	0.337±0.031 (n = 12)	0.814±0.020 (n = 14)	<0.0001

* , Pearson's correlation coefficients were determined using Zeiss Axiovision colocalization software and presented as mean ± S.E.

Diltiazem treatment significantly increased Pearson's correlation coefficients, indicating enhanced lysosome localization of GCase. Data were analyzed by Student's t test.

**Figure 4:**
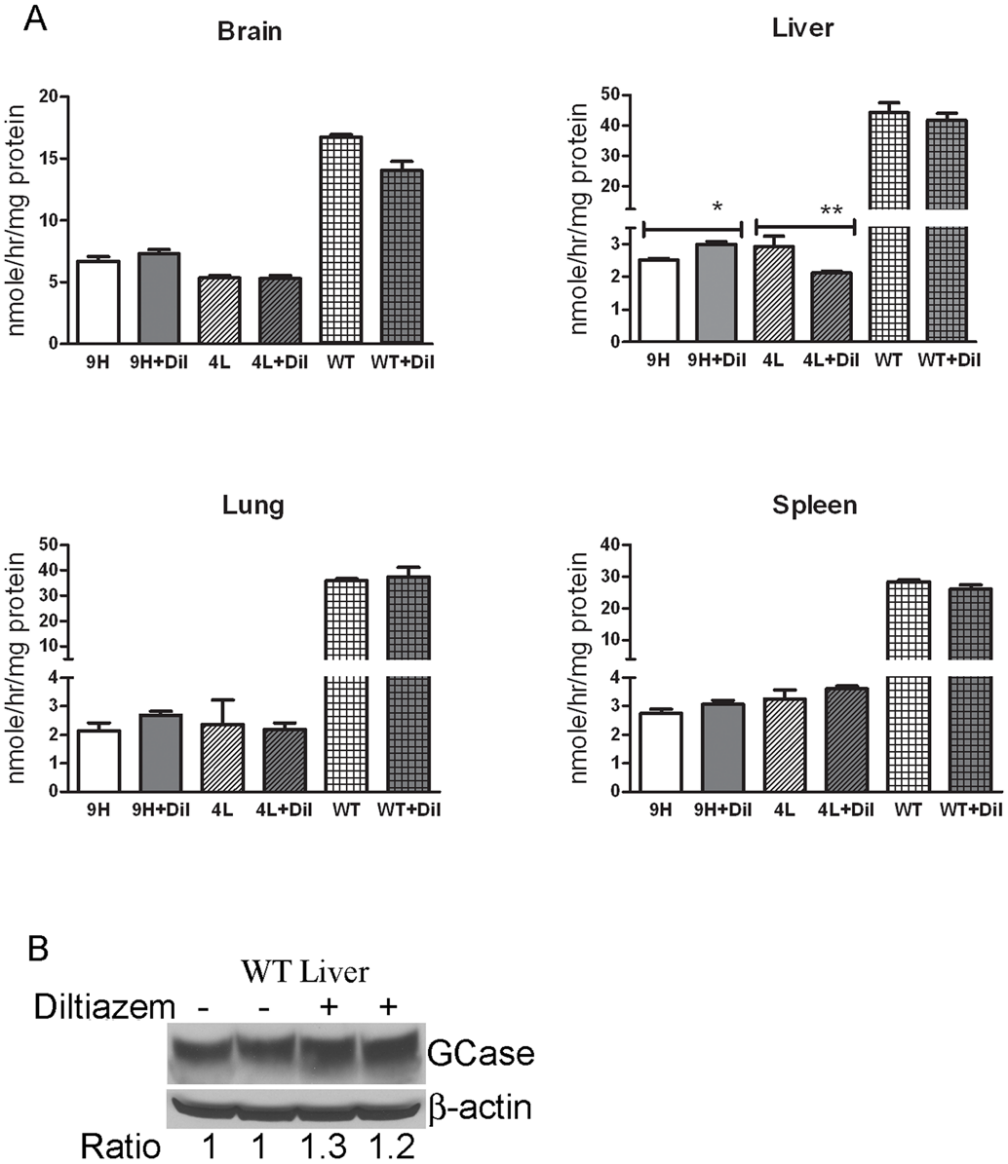
GCase protein and activity in diltiazem-treated mice: (A) The 4 wk old mice were administered diltiazem in drinking water (10mg/kg/day) for 4 wks. D409H livers had ∼1.2-fold increases of GCase activity. GCase activity in V394L liver decreased 38% after treatment. No significant changes in GCase activity were detected in WT, D409H and V394L brain, spleen, and lung. (B) Immunoblot analyses of GCase protein levels in WT liver. Diltiazem (10 mg/kg/d) increased GCase protein by ∼1.2 fold in WT liver. Fold change is presented as the ratio relative to untreated samples. Quantitation of blots was performed using Molecular Dynamics ImageQuant 5.0 Software and using the ratio of GCase/β-actin for normalization. Each experiment was repeated in triplicate. n = 3 to 7 mice. Student's t test, **, p<0.01; *, p<0.05.

To evaluate the *in vivo* effects of LTCC blockers on mutant GCase activity, diltiazem was administered to the mice since its effects in fibroblast cultures were more significant than those with verapamil. The doses were determined from the Pediatric Lexi-Drugs reference and from published studies on rodents [16], [17]. WT and GCase point-mutated (V394L and D409H homozygotes) mice at 4 wks were given diltiazem (10 mg/kg/d) in drinking water for 4 wks. GCase activities in the brain, lung, spleen, and liver were analyzed. The harvested tissues from the diltiazem-treated D409H mice showed a 1.2-fold increase in GCase activity in the liver relative to those in untreated mice (Fig. 4). V394L liver GCase activity decreased by 38% after treatment (Fig. 4). No significant change in GCase activity was detected in the brain, lung, and spleen of treated D409H and V394L mice or in any tissues from similarly treated WT mice (Fig. 4). GCase protein levels in livers from treated WT mice increased by ∼1.2-fold (Fig. 4B). In liver, variable increases (<1.2-fold) in the V394L and D409H GCase proteins were detected in treated mice (data not shown).

**Figure 5:**
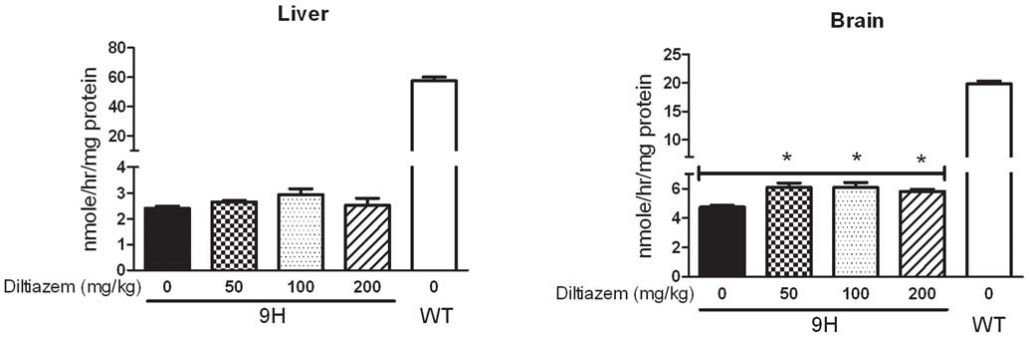
GCase activity in mice receiving diltiazem by intraperitoneal injection: The 9H mice were given diltiazem by intraperitoneal injections at 50, 100 or 200 mg/kg/d for 7 days. GCase activity increased by ∼1.2 fold in the brain of D409H mice at each of the three doses, i.e., the response was not dose dependent. No significant alterations of GCase activity were detected in the liver. Each experiment was repeated in triplicate. Untreated WT samples were also used as controls, n = 3 mice. Student's t test, *, p<0.05.

To ensure drug delivery and effective doses, additional two studies were conducted for 4 wks. Diltiazem (28 mg/kg/day) via drinking water was administered to D409H mice, and a higher dose of diltiazem (44 mg/kg/d) was given to V394L mice. The GCase activity in the brains and livers of these treated mice were not significantly altered compared to their untreated genotype-matched controls (data not shown). Additional D409H mice were given diltiazem by intraperitoneal injections with 50, 100 or 200 mg/kg/d for 7 days. These doses are ∼5–22 times greater than those (6–9 mg/kg/d) used in hypertensive patients [18]. After each injection the mice had very slow movements and major decreases in cage activity for several hours – an indication of toxicity. Full recovery to normal activity was evident by the next day. One mouse in the 200 mg/kg/d group died after the second injection. Small increases of brain GCase activities (∼1.2-fold) were detected in D409H mice receiving 50–200 mg/kg/d (Fig. 5). The effects on liver D409H GCase activities were minor. GCase activities were not altered in the lung and spleen of D409H mice (data not shown).

## Discussion

The LTCC blockers diltiazem and verapamil are approved by the FDA to treat hypertension and cardiac arrhythmias. They bind to LTCCs on the cell membrane and block calcium influx that could alter homeostasis in the ER [12], [18]. LTCC blockers enhanced mutant GCase activity and lysosomal targeting in fibroblasts from individuals with Gaucher disease and were suggested to have potential therapeutic use [12], [14]. The mechanism of how LTCC blockers alter GCase is not clear, but they appear to disrupt the unfolded protein response in these cells. Similar to the findings on human cultured fibroblasts [12], [14], analogous mouse cells carrying GCase point mutations showed increases in GCase protein and activity, and lysosome localization when cultured in the presence of diltiazem or varapamil. Diltiazem exhibited greater enhancements of the WT or mutant GCases than verapamil. However, neither diltiazem nor verapamil recovered GCase activity to heterozygote levels in the mutant cells. Curiously, the WT GCase activity in saposin C-deficient cells was increased by LTCC blockers. As one of its functions saposin C protects GCase from protease degradation in the lysosome [19]. Also, diltiazem inhibits the activity of the lysosomal nicotinic acid adenine dinucleotide phosphate (NADDP)-sensitive calcium release channel [20]. These findings imply additional effects of LTCC blockers at the lysosomal level that alter GCase stability directly in that organelle.

The bioavailability and drug metabolism of diltiazem have been studied extensively [18] and, importantly, diltiazem penetrates through the blood brain barrier [21]. In animal models, intraperitoneal, intravenous injections or oral administration of diltiazem in doses ranging from 10 to 200 mg/kg/d were effective in suppressing nicotine-induced place conditioning, protection of irradiation, and prevention of diastolic heart failure in a familial hypertrophic cardiomyopathy mouse model [16], [22], [23]. The doses of diltiazem used here (10–200 mg/kg/d) were greater than those used in a clinical setting to treat hypertension (6–9 mg/kg/d) in humans. At such high doses, the D409H mutant GCase activity was only incrementally enhanced in vivo.

The success of enzyme therapy (ET) for Gaucher disease has shifted the phenotype of the nonneuronopathic variant, type 1, toward normalcy in many patients [9]. However, the inability of macromolecules, like enzymes, to traverse the blood brain barrier (BBB) in sufficient amounts so to alter the CNS lysosomal storage has led to the search for alternative methods of treatment. Several small molecules have been the focus of interest, because they penetrate the BBB, modulate protein folding, and/or trafficking of endogenous mutant enzymes, e.g., GCase, to the lysosomes [12], [13]. The majority of these “chaperones” were evaluated in cultured cells, particularly fibroblasts from affected individuals [12], [24], [25], [26]. Such *ex vivo* studies provided data as proof-of-principle and suggest promise for their use as treatments for the neuronopathic variants of Gaucher disease. The therapeutic effects of these “chaperones” have not been tested in animal models with residual mutant GCase activity/protein to provide a physiological foundation for their potential clinical significance. Here, cells and tissues from our point-mutated GCase mouse models of GCase defective function were tested using LTCC blockers as potential therapeutic drugs. As shown in this study, diltiazem enhanced the activity and lysosomal trafficking of GCase in cultured fibroblasts from point mutated mice. *In vivo*, diltiazem had little if any effect on GCase activity or protein in WT and V394L mice. D409H fibroblasts had 1.8-fold enhancement of GCase activity by diltiazem. In D409H homozygous mice, the mutant GCase activity increase (∼1.2-fold) was not dose-dependent. This may be due to diltiazem having reached maximal effect *in vivo* at the lowest dose.

Thus, there is a discrepancy between the *in vivo* and *ex vivo* effects of diltiazem on GCase. The factors that contribute to these differential drug effects may include the effect of LTCC blockers on dividing (fibroblasts) versus non-dividing cells (neuron), the penetration efficiency of the drug into the ER (or lysosomes) in organs versus the cultured cells, and the distribution of LTCCs in different cell types and organs. The variation in expression of LTCCs on the membranes of microglial cells and macrophages is also impacted by various cellular states and microenvironments [27]. Mice with the most common human Gaucher disease mutations, N370S and L444P, have early lethality [8], [28], and thus, are not available for these studies. In addition, the alternative tissue specific knock-out models, the macrophage or neuronal GCase null mice [29], [30], would not be useful for these studies since residual GCase activity is required for LTCC blockers to have any effect. However, the D409H mutation is relatively common in the neuronopathic Gaucher disease variants [2], and, although rare, V394L has very similar kinetic and cellular properties, including response to LTCC blockers, as N370S. Thus, the use of LTCC blockers up to toxic doses in mice with such GCase mutations appears ineffective in significantly altering the mutant GCase activity or protein, suggesting that the LTCC blockers tested here would have no place in the treatment of individuals with Gaucher disease.

## Materials and Methods

### Ethics Statement

The mice were maintained in microisolators in accordance with institutional guidelines under IACUC approval at the Cincinnati Children's Hospital Medical Center.

### Cell culture and drug treatment

The mouse fibroblasts were derived from WT and point-mutated GCase mice including homozygotes for V394L, D409H, and N370S, the compound heterozygous D409V/N370S, homozygous selective saposin C deficiency (C−/−), and combined homozygotes for V394L and saposin C deficiency (4L;C*). The fibroblasts were established from the skin of one-day-old pups and cultured in DMEM with 10% fetal bovine serum, 1% penicillin, and 1% streptomycin. The cells were seeded at 35% confluence and incubated with diltiazem hydrochloride (Bedford Laboratories, Bedford, OH) or verapamil (Hospira, INC., Lake Forest, IL) at series concentrations for 5 days. The cells were harvested for analysis.

### GCase activity assay

The tissues were collected from saline-perfused mice. Fibroblasts and tissues were homogenized in 0.25% Triton X-100 and 0.25% sodium taurocholate in citric buffer, pH 5.6. GCase activities in the tissue and cell lysates were determined as described with 4-methylumbelliferryl β-D-glucopyranoside (4 MU-Glc, 4 mM) as substrate in 0.25% sodium taurocholate and 0.25% Triton X-100 [8]. Assay mixtures were preincubated in the presence and absence of the conduritol B epoxide (CBE, 1 mM), a specific irreversible inhibitor of GCase, for 30 min at 37°C. The substrate (4 MU-Glc) was added to the mixture, and incubated for 30 min at 37°C. Protein concentration was determined using the BCA protein assay reagent.

### Immunoblot Analyses

The tissue and cell lysates were prepared as described [31]. The cell (50 µg) and tissue (200 µg) lysates were resolved on Invitrogen NuPAGE 4–12% Bis-Tris gel with NuPAGE MED SDS running buffer and electro-blotted on Hybond^TM^-ECL nitrocellulose membranes. Rabbit anti-mouse GCase (1/1000 in 3% BSA) was used to detect mouse GCase, and mouse anti-β-actin monoclonal antibody (1/10,000) was used to detect β-actin. The signals were developed using an ECL reagent (Amersham Biosciences, Piscataway, NJ) according to the manufacturer's instructions.

### Fibroblast Colocalization Studies

Fibroblasts were treated with diltiazem at 25 µM for 5 days. LysoTracker Red (Molecular probe, L-7528) was applied to the V394L, WT, C−/−, and 4L;C* cells at 100 nM for 30 min at 37°C. The cells were rinsed with 1x PBS and fixed in 3% paraformaldhyde for 40 min at room temperature (RT). The fixed cells were quenched with 50 mM NH_4_Cl, rinsed with 1x PBS, and then treated with 0.5% saponin (Sigma, S-7900) and 1% BSA in 1 x PBS for 5 min at RT three times. The cells were incubated with rabbit anti-mouse GCase IgG (1/100) in 0.5% saponin-PBS, 1% BSA overnight at 4°C. D409H cells were incubated with rabbit anti-mouse GCase and rat anti-mouse Lamp1 (DRI, CD107a-D4B) (1/200) as a lysosome marker. The cells were washed with 0.5% saponin-PBS and labeled with goat anti-rabbit FITC (1/100) or goat anti-rat Texas red (1/100) for 1 hour at RT. Following a 0.5% saponin-PBS wash, the cells were mounted with mounting medium containing DAPI (Vector Laboratory). Fluorescence signals were visualized by Zeiss Axiovert 200 M microscopy equipped with an Apotome. Pearson's correlation coefficients were determined for individual cells using Zeiss Axiovision colocalization software. The data was analyzed by Student's t-test.

### Animal care and drug administration

The GCase point-mutated V394L and D409H homozygous mice were generated as described [8]. The strain background of V394L and D409H mice was C57BL/6J/129SvEV. WT mice are in FVB background. Drug administration was started in the mice at 4 wks of age. The first cohorts (homozygotes for V394L and D409H, and WT) were given diltiazem via drinking water at 10 mg/kg/d for 4 weeks. The dose in drinking water was determined using average daily water intake (5 ml) and mouse weight. The second cohort (D409H) was administered diltiazem by intraperitoneal injection at three doses: 50, 100, or 200mg/kg/d for 7 days. The third cohort, included D409H and V394L homozygotes, was given diltiazem in drinking water for 4 wks at 28 mg/kg/d for D409H homozygotes and 44 mg/kg/d for V394L homozygotes. The cage activities of the treated mice were monitored during the study.
